# Mesenchymal Stem Cells after Polytrauma: Actor and Target

**DOI:** 10.1155/2016/6289825

**Published:** 2016-06-02

**Authors:** Markus Huber-Lang, Rebecca Wiegner, Lorenz Lampl, Rolf E. Brenner

**Affiliations:** ^1^Department of Orthopaedic Trauma, Hand, Plastic and Reconstructive Surgery, University Hospital of Ulm, 89081 Ulm, Germany; ^2^Department of Anaesthesiology, Military Hospital Ulm, 89081 Ulm, Germany; ^3^Orthopedic Department, Division for Biochemistry of Joint and Connective Tissue Diseases, University of Ulm, 89081 Ulm, Germany

## Abstract

Mesenchymal stem cells (MSCs) are multipotent cells that are considered indispensable in regeneration processes after tissue trauma. MSCs are recruited to damaged areas via several chemoattractant pathways where they function as “actors” in the healing process by the secretion of manifold pro- and anti-inflammatory, antimicrobial, pro- and anticoagulatory, and trophic/angiogenic factors, but also by proliferation and differentiation into the required cells. On the other hand, MSCs represent “targets” during the pathophysiological conditions after severe trauma, when excessively generated inflammatory mediators, complement activation factors, and damage- and pathogen-associated molecular patterns challenge MSCs and alter their functionality. This in turn leads to complement opsonization, lysis, clearance by macrophages, and reduced migratory and regenerative abilities which culminate in impaired tissue repair. We summarize relevant cellular and signaling mechanisms and provide an up-to-date overview about promising future therapeutic MSC strategies in the context of severe tissue trauma.

## 1. Mesenchymal Stem Cells: A Multifaceted Adult Stem Cell Population

Mesenchymal stem cells, also referred to as multipotent mesenchymal stromal cells (MSCs), have first been isolated from bone marrow and characterized as a nonhematopoietic stem cell population with multilineage mesenchymal differentiation potential [1, 2]. Subsequently, cells with a MSC-like phenotype have been described in various neonatal (e.g., umbilical cord, placenta, and cord blood) and adult tissues (e.g., adipose tissue, synovial membrane, cartilage, bone, skin, muscle, liver, and lung) [3–6]. Functional characteristics of those cell populations seem to depend to a certain extent on the tissue source [5]. Moreover, MSCs have been attributed to a mixed developmental origin [6]. Since MSCs have received rapidly growing interest as a therapeutic tool or target in regenerative medicine the International Society for Cellular Therapy proposed the following minimal criteria for defining MSC: (1) adherence to plastic, (2) expression pattern of several surface markers (positive: CD73, CD90, and CD105; negative: CD45, CD34, CD14 or CD11b, CD79alpha or CD19, and HLADR surface molecules), and (3) osteogenic, adipogenic, and chondrogenic differentiation potential [7]. These minimal criteria clearly define heterogenous cell populations with widespread distribution in the body [5]. However, they have been used in most studies so far. In a more stringent sense, CD146-positive subendothelial cells from bone marrow have been proposed as clonogenic, self-renewing multipotent skeletal stem cells which also support hematopoiesis [8]. Besides high proliferation capacity [1] and migratory activity in response to chemoattractive factors [9] the differentiation potential into various mesenchymal lineages such as osteoblasts, chondrocytes, adipocytes, tenocytes, and muscle cells [10] or a certain transdifferentiation capacity [11], for example, into neural cell types [12] or hepatocytes [13], attracted much interest in the context of regenerative medicine. The original concept was that MSCs could regenerate tissues by engraftment and differentiation into the respective tissue-specific cell types. Later it was recognized that MSC could additionally support regenerative processes by secretion of trophic factors and by immunomodulatory activity [14–16]. The relative contribution of these synergistic functionalities is not clearly defined so far and may depend on the origin of the involved MSC population, the respective target tissue, the severity and kind of tissue damage, and the extent of local and systemic inflammatory reaction.

## 2. Polytrauma: A Multifaceted Challenge

Polytrauma has been defined as two or more injuries (multiple injuries) with at least one injury or the sum of all injuries being life-threatening [17]. The pathophysiological consequences of polytrauma are extremely complex and do not reflect the sum of all separate injuries but rather a unique global amplified challenge of all organs [18]. Even remote tissues which were primarily not injured become affected by the systemic danger response to various pathogen- and danger-associated molecular patterns often resulting in systemic inflammatory response syndrome (SIRS), sepsis, and finally multiple-organ dysfunction syndrome (MODS) endangering life a second time. Thus, polytrauma may in principal transform any organ and single cell into “actors” driving the danger response* after trauma* and thereby adding to tissue damage proposed as “second hit.” Subsequently, all cells may theoretically also transmogrify to a “target” of the general danger response, in particular* per* the inflammatory reaction, coagulatory response, complement attack, oxidative burst reaction, bacterial invasion, and so forth [19]. The multifaceted cellular response to polytrauma also includes cells with a physiologically high regenerative potential such as MSCs. After severe trauma MSCs may be challenged by the balancing act between cellular recruitment and immunomodulation to promote healing versus inactivation and death with resulting impairment or absence of sufficient healing. Although clinical data are rare, there is growing experimental evidence that the relative contributions of these MSC functions are critical for understanding the role of MSCs in mediating recovery (or the lack thereof) in the context of polytrauma.

## 3. Recruitment of MSCs after Polytrauma

MSCs are crucial for the initiation of regenerative processes. Inconsistent numbers of circulating cells have been detected in experimental and clinical trauma settings [20–23], and their homing behaviour to bone marrow or migration to damaged tissue remains elusive. Furthermore, bone marrow-derived MSCs revealed enhanced proliferative capacity which was somehow dependent on the severity of trauma [24]. The trauma-triggered mobilization of MSCs from the bone marrow can be caused by hypoxia [25], various danger-associated molecular patterns (DAMPs, e.g., histones and mitochondrial debris), and chemoattractants (e.g., [26]), all of which are generated after severe injury. When synchronically exposed to key mediators of the trauma response, such as IL-1*β*, IL-6, IL-8, C3a, and C5a (in concentrations corresponding to those measured in the blood of polytrauma patients), MSCs exhibited an increased chemotactic activity. Particularly the central complement activation product C3a was able to remarkably enhance their migratory activity [27]. Similarly, the anaphylatoxin C5a has been found to be a chemoattractant for MSCs in higher concentrations [28], implying that complement activation at the injury site may result in a strong chemotactic signal for MSC recruitment. However, other established factors also enable MSCs to migrate towards the place of injury: they have been shown to relocate to fracture sites target-specifically in response to soluble mediators including the chemokine stromal cell-derived factor-1 (SDF-1) [29]. Granulocyte colony stimulating factor (G-CSF) represents another potent MSC mobilization factor. In patients with severe trauma, G-CSF has recently been demonstrated to be upregulated more than 50-fold and even higher in case of an additional hemorrhagic shock [30]. In turn, G-CSF may not only mobilize MSCs but also induce a bone regenerative response, for example, by an increased expression of bone morphogenetic protein-2, growth differentiation factor-9, IL-10, IL-8, and nodal growth differentiation factor, as recently shown* in vitro* [31]. During neurotrauma, lysophosphatidic acid (LPA), a bioactive phospholipid, has been demonstrated to play a causative pathophysiological role [32]. Interestingly, LPA is also known to be an effective mobilizer of MSC [33]. Further inflammatory mediators generated after polytrauma [19], such as tumor necrosis factor (TNF), macrophage migration inhibitory factor (MIF), and extracellular HMGB-1 (high mobility group box 1) as a key DAMP, are potent recruiters for MSCs to the site of injury [33].

It is noteworthy, however, that almost all tissues are home to residential MSC-like cells which after infliction of injury may initiate tissue regeneration independently of or even despite additionally recruited MSCs. In this regard, a recent study was unable to detect MSCs in the human blood circulation under conditions such as end-stage renal or liver disease or during heart transplant rejection and thus proposed that bone marrow disruption caused by multiple fractures rather than solid organ injury may be the reason for MSCs to appear in the circulation [21].

It is crucial that MSCs are not only mobilized to injured tissue, but also able to adequately differentiate upon arrival. However, MSC differentiation mechanisms after polytrauma are rarely investigated. We and others have proposed C5a-C5a receptor (C5aR) interactions to be involved in osteogenic differentiation since C5aR was increasingly expressed as human MSCs differentiated to osteoblasts [34, 35]. Furthermore, the altered C5aR expression profile upon differentiation was strongly dependent on the urokinase receptor (uPAR) and NF-*κ*B pathway, indicating that the uPAR-C5aR-NF-*κ*B signaling cascade controls osteogenic differentiation in MSCs [35]. Apart from MSCs, CD34-positive progenitor cells are also considered competent in osteogenic and endothelial differentiation, and their numbers in circulation have also been reported to be increased up to 7 days after severe trauma [36].

## 4. MSCs as Actors after Trauma

**Figure 1:**
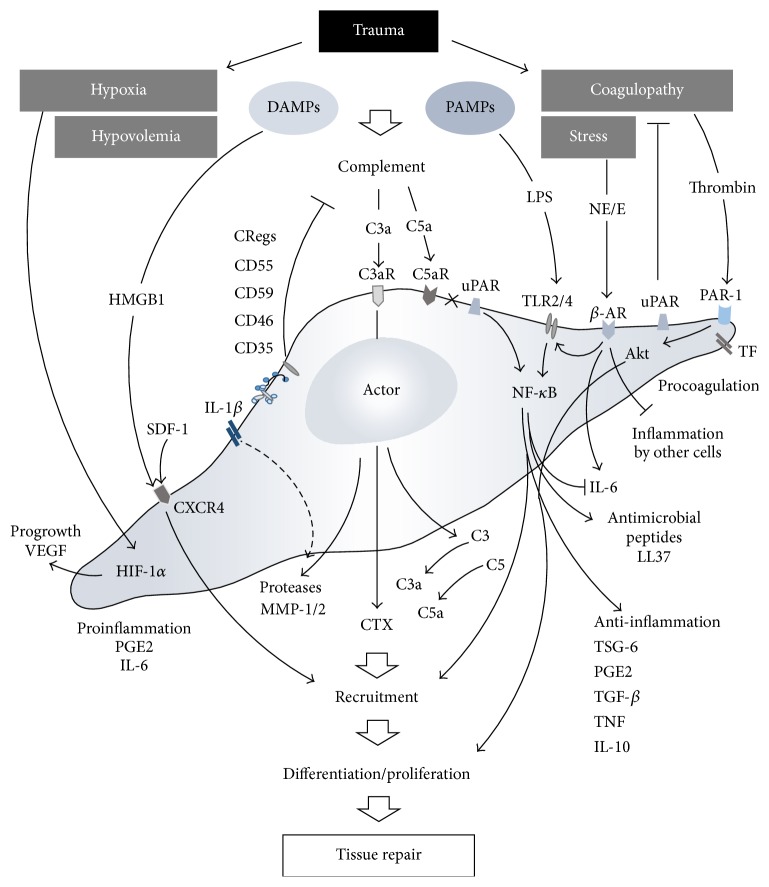
After trauma, MSCs are challenged with local and systemic hypoxia, hypovolemia, disturbances in coagulation, and released danger molecules, inducing them to act as mediators in vast numbers of processes and ideally contributing to successful tissue repair. C3aR: complement C3a receptor; C5aR: complement C5a receptor; CRegs: complement regulatory proteins; CTX: chemotaxis; CXCR4: C-X-C chemokine receptor type 4; DAMPs: damage-associated molecular patterns; HIF-1*α*: hypoxia inducible factor-1 alpha; HMGB-1: high mobility group box 1; IL: interleukin; LPS: lipopolysaccharides; MMP: matrix metalloproteinase; NE/E: norepinephrine/epinephrine; *β*-AR: beta-adrenergic receptor; NF-*κ*B: nuclear factor kappa-light-chain-enhancer of activated B cells; PAMPs: pathogen-associated molecular patterns; PAR-1: protease-activated receptor 1; PGE2: prostaglandin E2; SDF-1: stromal cell-derived factor-1; TF: tissue factor; TGF-*β*: transforming growth factor beta; TLR: toll-like receptor; TNF: tumor necrosis factors; TSG-6: tumor necrosis factor-inducible gene 6 protein; uPAR: urokinase receptor; VEGF: vascular endothelial growth factor.

Regardless of their origin, migrated and resident MSCs are thought to sustainably modulate the local and systemic inflammatory response after trauma and to induce and control the regenerative processes in damaged tissue (Figure 1). The main character of MSCs after trauma appears multifaceted and may include growth-enhancing, antiapoptotic, anti-inflammatory, antioxidative, antimicrobial, and other features as recently comprehensively reviewed for single acute organ injuries [37]. However, in the context of combined trauma (e.g., tissue trauma plus hemorrhagic shock) and polytrauma, there is still uncertainty of how MSCs act.

It is established that the MSCs are potent anti-inflammatory actors. In experimental polytrauma, bone marrow MSC application inhibited LPS-associated acute lung injury (ALI) and underlying TLR2/4 upregulation within the lungs and remarkably shifted the proinflammatory cytokines towards an anti-inflammatory cytokine profile [38].

Exposure of MSCs to IL-1*β* concentrations found in serum early after polytrauma resulted in generation and release of metalloproteinase-1 (MMP-1), tumor necrosis factor-inducible gene 6 (TSG-6), cyclooxygenase-2, and prostaglandin E synthase, all of which act as key immunomodulators of the posttraumatic response [27]. Furthermore, IL-1*β*-triggered TSG-6 generation by MSCs may switch the proinflammatory M1 macrophage phenotype towards the rather anti-inflammatory M2 macrophage phenotype and thereby improve wound healing [39].

Polytrauma-induced massive activation and subsequent dysfunction of the coagulation and complement system [40] may also determine MSC behaviour. Thrombin as a central coagulation molecule in the activated clotting cascade after polytrauma results in expansion of MSCs via protease-activated receptor- (PAR-1-) mediated Akt signaling and subsequent robust upregulation of c-MYC [41]. When exposed to the key activation product of the related complement system, C3a, in concentrations measured early after multiple injuries, MSCs significantly upregulated angiogenic factors such as vascular endothelial growth factor (VEGF), CXCL8/IL-8, but also IL-6. In turn, these factors induced* in vitro* minimal tube formation of endothelial cells indicative of angioneogenesis [42].

Bone marrow-derived MSCs also exhibit innate procoagulatory activity most likely based on the expression of tissue factor (TF) on MSCs, resulting in increased clotting, decreased fibrinolysis, and microvascular obstructions [43] which may reflect conditions found in advanced stages of acute trauma-induced coagulopathy. Concerning platelets within the clotting process, platelet-derived growth factors (PDGF) and other platelet-originated products are able to induce MSCs expansion* ex vivo*. In the setting of severe trauma, serum PDGF-AA and PDGF-BB levels were associated with the number of MSCs obtained from the bone marrow of the injured patients [23]. Contrary to other reports, that study failed to show a significant increase in bone marrow homing of MSC, nor could a significant recruitment of MSCs into the peripheral blood be observed after severe injury, irrespective of the trauma severity. Nevertheless, serum from polytrauma patients induced MSC proliferation in a PDGF-associated manner [23].

Concerning complement generation, MSCs do in fact express various complement receptors, such as C3aR and C5aR, [44] by which they are able to sense chemotactically active anaphylatoxins. Furthermore, MSCs are also capable of generating key complement components, such as C3 and C5 [34], and thus after cleavage by various activated coagulation factors may generate the potent anaphylatoxins C3a and C5a, both of which can induce all classical signs of local and systemic inflammation found after severe tissue injury. Indeed, MSCs were found as a complement activator upon exposure to ABO-matched human blood resulting in production of C3a which in turn governs the immunomodulatory features of MSCs and the interactions with other immune cells [45].

As further action mechanisms of MSCs* after injury*, hypoxia during trauma-hemorrhagic shock not only may support preservation of undifferentiated MSCs but also may increase their regenerative potential and moreover may activate hypoxia inducible factor-1 (HIF-1) in MSCs which in turn results in an increased expression of VEGF for neovascularization [25].

Whether all these effects of MSCs are due to the direct cellular actions, the secretion of cytokines, or (in part) microvesicles shed from MSCs is unknown. Extracellular MSC vesicles have been shown to protect against hypoxia-induced acute kidney injury. Interestingly, when the MSC-derived vesicles were generated in a simulated inflammatory micromilieu, the microvesicles containing tetraspanins failed to reverse the kidney injury. In contrast, effective microvesicles originated from otherwise untreated MSCs contained the complement factors C3, C4A, and C5 [46] which may assist in further cell recruitment and induction of regeneration processes [47, 48].

Paracrine and endocrine functions of MSCs have recently been more and more in the focus of research [49]. Besides the inflammation-modulatory functions, MSCs seem also to influence endothelial and epithelial permeability resulting in an enhanced clearance of alveolar fluid [50]. This may be of particular importance for polytrauma-induced blood-organ barrier dysfunction and associated multiple-organ dysfunction syndrome. In this context, in both murine polytrauma model and polytrauma patients, we have recently shown evidence of the tight junction molecule, junctional adhesion molecule-1 (JAM-1), circulating in the blood [51]. In a rodent ischemia-reperfusion (I/R) injury model of the superior mesenteric artery, bone marrow-derived MSCs acted as inhibitors of zonula-occludens-1 (ZO-1) downregulation and tight junction disruption via a TNF-controlled mechanism [52]. These observations support the idea of MSCs improving crucial cellular barrier functions after severe tissue trauma.

## 5. MSCs as Targets after Trauma

**Figure 2:**
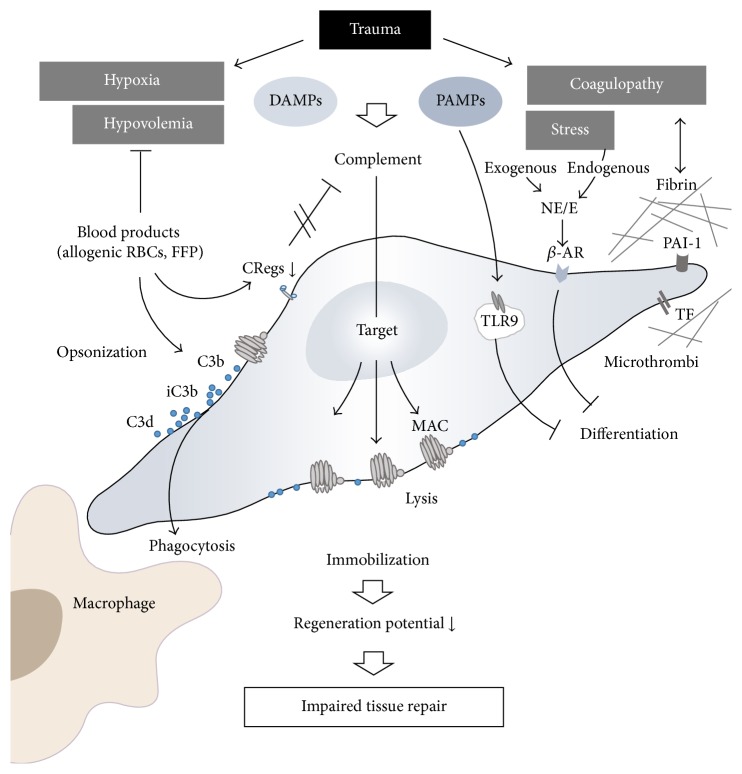
MSCs also function as targets of pathophysiological processes after trauma, leading to complement opsonization and macrophage phagocytosis and reduction in differentiation potential or ability to migrate to the site of injury and finally resulting in the impairment of regenerative potential and tissue repair. See text for detailed information. CRegs: complement regulatory proteins; DAMPs: damage-associated molecular patterns; FFP: fresh frozen plasma; MAC: membrane attack complex; NE/E: norepinephrine/epinephrine; *β*-AR: beta-adrenergic receptor; PAI-1: plasminogen activator inhibitor 1; PAMPs: pathogen-associated molecular patterns; RBC: red blood cells; TF: tissue factor; TLR9: toll-like receptor 9.

Besides their function as “activators” and “suppressors” of the systemic inflammatory response* after trauma*, MSCs are equipped with a broad arsenal of defense mechanisms against immunological attacks. Thus, they seem to present an important “target” cell for the immune system after multiple injuries (Figure 2).

The procoagulatory surface mainly formed by tissue factor (TF) expression on MSCs [43] renders these cells as potential focal points of fibrin generation and subsequent effective cellular immobilization. This process might also be supported by expression of the plasminogen activator inhibitor 1(PAI-1) on MSCs [53]. As a potential defense mechanism against this fibrin “cladding,” fibrinolytic factors (e.g., uPAR) are expressed on MSCs [53] which in concert with various released proteases may dissolute any fibrin thrombi.

The MSCs represent a major target for complement attacks. Abundant deposition of the C3 fragments iC3b and C3dg on MSCs and thus opsonization of the MSCs exposed to ABO-matched allogenic human blood have been found [45]. To counteract a harmful complement attack and opsonization MSCs express a remarkable variety of membrane bound complement regulatory proteins (CRegs), such as protectin (CD59), decay accelerating factor (CD55), and membrane cofactor protein (CD46) [34]. Furthermore, MSCs also release factor H which results in direct inhibition of C3 cleavage and opsonization [54]. However, despite these potent complement inhibitory strategies, contact of MSCs with serum (e.g., provided by massive transfusions after polytrauma) may overwhelm these defense mechanisms and result in serum-induced cytotoxicity [55]. Experimentally, adoptively transferred MSCs in mice deficient in C3 or in mice after C3 depletion (by cobra venom factor) exhibited significantly reduced MSC injury* in vivo* compared to MSCs in wildtype mice [55]. These findings indicate that complement inhibitory strategies in MSCs are crucial for survival and regenerative potential of these cells after trauma. All of the abovementioned CReg proteins on leukocytes are somehow dysregulated early after polytrauma in humans [56]. Possibly, also on MSCs, the CReg shield might be disturbed after multiple injuries and therefore may turn MSCs into targets for a fatal complement attack.

Circulating histones and mitochondria have been identified as DAMPs in patients after severe tissue injury [57–59], inducing a robust inflammatory response. Furthermore, MSC fate determination including differentiation seems to be crucially dependent on histone-modifying enzymes and various transcription factors [60]. Thus, it is tempting to speculate that polytrauma conditions may manipulate histone signatures and thereby disturb regenerative potential of MSCs. However, further research has to elucidate underlying mechanisms.

Exposure to trauma-released mitochondria, mitochondrial DNA, and debris [59] leads to toll-like receptor (TLR) activation in MSCs which in turn may result in an antagonization of MSC differentiation into a specific tissue [61]. Thus, mitochondrial DAMPs may significantly alter MSC proliferation and differentiation and may affect MSC multipotency [61], finally leading to an impaired or altered regeneration after severe tissue trauma.

It is important to consider that, directly after polytrauma, there is a strong stress reaction resulting in an extensive release of endogenous catecholamines, including epinephrine and norepinephrine. Interestingly, activation of the corresponding *β*-adrenoreceptor on MSCs leads to inhibition of their differentiation potential [62]. To what extent additionally applied exogenous catecholamines (e.g., norepinephrine), given to stabilize hemodynamic function, will compromise tissue regeneration by suppressing MSC function or differentiation is of great clinical interest and needs to be clarified in future translational studies.

## 6. Therapeutic Potential of MSCs in Polytrauma 

### 6.1. Current Challenges

A major challenge is the transfer of the numerous* in vitro* findings of multifaceted MSC functions to relevant and reliable preclinical studies and finally the translation to the clinical setting. The optimal MSC source (e.g., bone marrow, adipose tissue, and umbilical cord), the timing after trauma, the administration route, and number of applied cells remain to be defined for the polytrauma situation. In addition, possible immunosuppressive functions of MSC in a polytrauma-induced compromised immunological situation may increase the risk of life-threatening infections. Noteworthily, the acute trauma situation does not allow time and tissue consuming procedures for cell isolation, characterization, and expansion rendering an autologous MSC transplantation strategy questionable. Furthermore, socioeconomic considerations with high logistic demands (inclusive GCP/GLP-conform MSC preparation), high costs, and high variability of the individual injury pattern currently prevent a broad therapeutic platform for MSC in polytrauma patients.

### 6.2. Progress Made

Nevertheless, various preclinical studies have already addressed the therapeutic potential of MSC in single injury models of different tissues and organs [37, 63]. These experimental approaches include physical trauma of the skin [39, 64], muscle [65], skeletal tissues [66, 67], lung [68, 69], brain [70–72], and spinal cord [73], all of which are frequently affected in polytrauma patients (Annual Report 2013, TraumaRegister DGU®). Moreover, their therapeutic effect in specific pathophysiological situations frequently developing in polytrauma patients, for example, sepsis [74–76], has been studied. In most cases, the therapeutic strategies were based on the concept of MSCs as “actors” delivered by local or systemic cell transplantation. The majority of these studies on monotrauma models indicated therapeutic benefits, although the absolute number of transplanted cells systemically recruited to the site of the injury or surviving in injured regions after local injection was rather low. Therefore, reported therapeutic effects were mainly attributed to the release of trophic factors and immunomodulation [14]. In mice, systemic application of allogeneic MSCs leads to limited local recruitment and stimulation of bone formation assessed by *μ*CT analysis in a fracture model while it had no additive effects on bone formation induced by repetitive mechanical stimulation [67]. This indicates that the trauma situation, most probably the posttraumatic inflammatory reaction, triggers this functionality. Since the respective environment is greatly dependent on the extent and combination of different traumatic injuries, the situation in a polytraumatized patient may be quite different. So far, only few studies addressed this highly relevant clinical situation. Thorax trauma occurs frequently in combination with other injuries and is highly relevant for the polytrauma mortality. Interestingly, chest trauma also influences the course of other injuries like fracture healing in rats [77, 78]. On the other hand, in the same species, the resulting histologic lung alteration is aggravated by parallel hemorrhagic shock or chronic stress. Systemic infusion of allogenic MSC in male rats reduced the lung injury score after lung contusion with hemorrhage or chronic stress [79, 80] and restored the disturbed bone marrow function characterized by reduced clonal growth of bone marrow cells and persistent anemia [79, 81]. In these models, MSC application also increased the relative amount of regulatory T cells [79, 80]. Even in the most compromised situation combining lung contusion, hemorrhagic shock, and chronic stress, the MSC therapy proved to be effective [82]. Since this situation more closely resembles the polytrauma setting in human patients, a therapeutic benefit through future application of MSCs can be expected. In another study where multiple fractures were combined with hemorrhagic shock in rats, systemic MSC application improved weight gain, physical activity, muscle atrophy, and fracture callus histology [83]. In the polytrauma situation, due to vascular damage and hypotension, prolonged ischemia of various organs may be another critical factor. In this context it could be shown that MSC treatment attenuated lung I/R injury in rats [84]. Furthermore, in a mouse model, intravenously applied allogeneic MSCs protected lung transplants from cold I/R injury [85]. In this study, the cell-therapeutic effects were associated with reduced cellular apoptosis, decreased infiltration of macrophages, neutrophils, and CD8+ cells, and lower amounts of TNF, IL-6, and TLR4 but higher expression of TSG-6, in lung tissue [85]. Most of the previously mentioned* in vivo* studies concentrated on major clinically relevant outcome parameters and not on underlying molecular processes. Based on the current knowledge in this field, it could be speculated that a combination of different processes might be involved as illustrated in Figures 1 and 2. Only in some studies on monotrauma models the presence of transplanted cells is documented in the injured tissue. Whether local recruitment and simply survival of transplanted cells are determining factors in regeneration after multiple injuries is not known so far.

### 6.3. Current Limitations

Numerous clinical trials are currently under way but only a very limited number address acute physical trauma situations [86]. As recently reviewed by Squillaro et al., 493 MSC-based clinical trials are currently listed in the National Institute of Health database, addressing various areas such as graft-versus-host disease, hematological disease, diabetes, organ transplantation, and inflammatory diseases [86]. Only two studies address acute lung injury [37], and, to our knowledge, no study has focussed on the polytraumatized patient. As mentioned above, due to the clinical situation and critical timing including limited time for autologous MSC expansion, polytraumatized patients would require allogenic application of MSCs in future studies. This may theoretically be feasible since allogeneic applications have already been performed in refractory lupus erythematosus patients and in steroid-resistant graft-versus-host disease patients without serious adverse effects [87, 88]. Furthermore, only limited information is available about differential immunosuppressive functionality [19, 89] as well as spatial and temporal regenerative potential of MSCs originated from different tissues. Consequently, great caution is necessary in clinical translation of experimental findings defining MSCs as “actors” and “targets” since MSCs resident in different tissues, MSCs mobilized after trauma, and MSCs after* ex vivo* expansion and transplantation may not function identically and thus may not be interchangeable.

### 6.4. Future Directions

A promising approach to address the therapeutic potential of MSC would be the injection of factors that systemically mobilize or locally target endogenous stem cells. Such a strategy was reported by Hannoush et al. for acute physical lung injury in male rats [68]. Systemic G-CSF application for 5 days prior to lung contusion leads to an increase of hematopoietic progenitor cell colony growth in the traumatized lung [68]. However, the question of whether MSCs were also systemically mobilized remained open in this study. Nevertheless, the resulting lung injury score was improved by G-CSF pretreatment and by SDF-1 injection into the lung (or by the combination of both) similarly to the effects seen after systemic application of allogeneic MSCs [68]. Strategies addressing the local recruitment of MSCs to date mainly investigate CXCR4 activation by SDF-1 [90]. As a future therapeutic avenue, modulation of the activated complement system may also support endogenous MSC recruitment since the anaphylatoxins C3a and in higher concentrations also C5a stimulate directed MSC migration as mentioned earlier [27, 28, 44, 91]. Noteworthily, in severe trauma situations, catecholamines via induction of genes involved in migration may support mobilization of MSCs [19, 92]. On the other hand, catecholamines were reported to inhibit differentiation into adipogenic, osteogenic, and chondrogenic lineage which may reflect differential activity of MSCs depending on the functional demand [92]. In addition, MSCs are able to inhibit the inflammatory response of other cells such as macrophages [93].

Micro-environment-tailored strategies to improve engraftment at the lesion site may include preconditioning with cytokines or growth factors, platelet-enriched plasma, complement regulators, hypoxia, genetic modifications, or modification of MSC surface structures with antibodies or coating with homing ligands [94–96]. Also, improving the survival of transplanted cells in a compromised milieu, for example, by hypoxic preconditioning in I/R injury in rats [84, 97] may offer the chance to further increase the therapeutic potential and to reduce the rather high numbers of cells that are usually applied. Immunoselection based on expression of specific functional markers reflects a further important strategy to direct cells to the insulted region of interest. This has recently been shown for selected CXCR4-positive MSCs, revealing a significantly improved migratory and healing profile and remarkable synchronic suppression of the systemic inflammatory reaction [29]. Other treatment strategies with the MSC secretome or MSC microvesicles have not yet been tested in the setting of multiple trauma. Nevertheless, they may be promising based on observations on other disease models [98, 99].

## 7. Conclusion

Numerous* in vitro* and* in vivo* observations clearly indicate that MSCs are central players in the complex network of pathophysiologic events after major trauma. Many questions, however, still remain open in order to therapeutically address MSCs as either “actors” or “targets” in the polytrauma setting. These include the optimal cell source (e.g., bone marrow, adipose tissue, and umbilical cord), the timing and balancing in the posttraumatic scenario of pro- and anti-inflammatory reactions, the application route and dosage of cells, and possible immunosuppressive functions of MSC in a compromised situation carrying the danger of life-threatening infections. Future translational studies are needed to answer these questions and to individually and beneficially utilize the ambivalent and multifaceted behaviour of MSCs.
